# Air ions and respiratory function outcomes: a comprehensive review

**DOI:** 10.1186/1477-5751-12-14

**Published:** 2013-09-09

**Authors:** Dominik D Alexander, William H Bailey, Vanessa Perez, Meghan E Mitchell, Steave Su

**Affiliations:** 1 Exponent, Health Sciences, Boulder, CO, USA; 2 Exponent, Health Sciences, 17000 Science Drive, Suite 200, Bowie, MD 20715, USA; 3 Exponent, Health Sciences, Chicago, IL, USA; 4 Exponent, Health Sciences, New York, NY, USA

## Abstract

**Background:**

From a mechanistic or physical perspective there is no basis to suspect that electric charges on clusters of air molecules (air ions) would have beneficial or deleterious effects on respiratory function. Yet, there is a large lay and scientific literature spanning 80 years that asserts exposure to air ions affects the respiratory system and has other biological effects.

**Aims:**

This review evaluates the scientific evidence in published human experimental studies regarding the effects of exposure to air ions on respiratory performance and symptoms.

**Methods:**

We identified 23 studies (published 1933–1993) that met our inclusion criteria. Relevant data pertaining to study population characteristics, study design, experimental methods, statistical techniques, and study results were assessed. Where relevant, random effects meta-analysis models were utilized to quantify similar exposure and outcome groupings.

**Results:**

The included studies examined the therapeutic benefits of exposure to negative air ions on respiratory outcomes, such as ventilatory function and asthmatic symptoms. Study specific sample sizes ranged between 7 and 23, and studies varied considerably by subject characteristics (e.g., infants with asthma, adults with emphysema), experimental method, outcomes measured (e.g., subjective symptoms, sensitivity, clinical pulmonary function), analytical design, and statistical reporting.

**Conclusions:**

Despite numerous experimental and analytical differences across studies, the literature does not clearly support a beneficial role in exposure to negative air ions and respiratory function or asthmatic symptom alleviation. Further, collectively, the human experimental studies do not indicate a significant detrimental effect of exposure to positive air ions on respiratory measures. Exposure to negative or positive air ions does not appear to play an appreciable role in respiratory function.

## Introduction

Over the past 80 years, extensive literature has been published pertaining to the potential biological effects of air ions. One of the major topics within this literature concerns the effect on respiratory function and health consequences, both favorable and unfavorable, after exposure to ionized air [1-3]. Small air ions are electrically charged clusters consisting of atmospheric molecules or atoms that have lost or gained electrons to impart a net positive or negative charge [4]. Atmospheric space charge in the form of small air ions may be generated from natural sources, such as changes in atmospheric and weather conditions, including rain, wind, and snow, as well as natural radioactivity in geological formations, cosmic radiation, waterfalls, and combustion processes [4]. In addition, air ions are produced by air ionizer devices sold to clean indoor air of aerosols and particulate by electrostatic precipitation; they also are produced by corona activity on the surface of high voltage transmission conductors of alternating current (AC) and direct current (DC) transmission lines. Scientists and meteorologists have measured naturally occurring variations of the electrical charge in the air for more than 100 years [5].

Historically, a variety of physiological or health effects in relation to exposure to charged air ions have been suggested. In general, many researchers have indicated a beneficial or therapeutic effect on lung function, metabolic measures, and asthmatic symptoms after exposure to negative air ions [6-8]. In contrast, a few researchers have suggested that exposure to positively charged air ions may be associated with decreased pulmonary performance and may exacerbate asthmatic symptoms and other respiratory irritations [8-10]. Yet, the constellation of scientific evidence relating to either a beneficial or detrimental respiratory effect after exposure to charged air ions remains unclear and has not been systematically reviewed in the past 30 years. Further, there is skepticism that concentrations of air ions in the range of 100,000 ions/cm^3^ (i.e., 1^5^/10^-19^), for example, would have biological effects at concentrations similar to one of the most toxic chemicals (e.g., botulism at 10^-14^) [11]. The published scientific studies on this topic span over 80 years, and vary by differences in research methodology, clinical and laboratory technology, statistical techniques and capabilities, study population dynamics, and changes in environmental factors.

Although published research on air ion exposure and respiratory outcomes span numerous decades, to our knowledge, there are no current reviews on this topic, aside from a recent Cochrane Collaboration evaluation of air ionizers and asthma, for which the researchers did not recommend the use of room air ionizers to reduce symptoms in patients with chronic asthma [1]. Thus, our objectives were to summarize and review the published human experimental studies of exposure to negatively and positively charged air ions and respiratory function and outcomes, such as clinical pulmonary measures and asthmatic symptoms. In addition, where appropriate, we quantified results for similar exposure and outcome groupings using meta-analytic methods and forest plot illustrations of the data.

## Materials and methods

### Literature search and study identification

A structured literature search was conducted to identify the cumulative literature on the effects of charged air ions on acute and chronic respiratory function measures in humans. An earlier comprehensive review of possible biological and health effects of DC transmission lines commissioned by the Minnesota Environmental Quality Board [12] was used to identify the relevant historical literature through 1982. To update and supplement these earlier studies, a literature search using the Medline (PubMed) bibliographic database was conducted to identify articles indexed between January 1, 1982 and July 1, 2011. The DIALOG search service also was used to retrieve studies from relevant life, environmental and behavioral sciences, engineering, and other technical databases, including El Servier Biobase and Embase. Both the PubMed and DIALOG searches employed the same search strings. For our PubMed search, we used Medical Subject Heading (MeSH) terms for air ionization, which yielded 518 articles. We then incorporated terms in the title and abstract which referenced the exposure (air ions, charged aerosols, corona ions, atmospheric ions, ionization, ionized air, heavy ions, or light ions) and outcomes (respiration, asthma, lung cancer, chronic obstructive pulmonary disease, allergy, or rhinitis) of interest. The literature review was supplemented by hand searching the reference lists of all retrieved studies on this topic. In addition, we checked the recent Cochrane Collaboration [1] review on ionizers and chronic asthma to identify English language studies^1^.

Articles were restricted to studies among human populations published in the English language. We included experimental studies of subjects exposed to negatively or positively charged (or both) small air ions in a controlled or uncontrolled environment. Specifically, studies were required to report exposure to air ions with respect to their relationship (typically involving data on individual or group mean comparisons) on respiratory function outcomes (e.g., forced expiratory volume [FEV]), metabolic or other physiologic measures (e.g., blood pressure), or asthmatic or subjective symptoms (e.g., wheezing). We excluded studies for which only fitness or physical performance was evaluated (unless data on respiratory function was documented), and we excluded articles based on human survey data as well as experimental studies of animals and isolated cells and tissues. No restrictions on the number of subjects evaluated in each study were imposed because of the wide variation in publication dates and experimental methodologies. Twenty-three studies, published between 1933 and 1993, on the acute and chronic respiratory effects of air ions were identified that met our inclusion criteria. Some non-respiratory outcomes of air ion exposure were described in these 23 studies and these outcomes were also reviewed to insure that any potentially relevant related effects were not overlooked.

### Data extraction and statistical methods

Qualitative information (e.g., characteristics of study population, study design) and quantitative data (e.g., group mean data for peak expiratory flow rate [PEFR], changes in blood pressure) were extracted and tabulated from each experimental study that met the inclusion criteria for further review (Table 1). Studies varied by population characteristics, evaluation of ion polarity, and outcome measures. Thus, in an effort to harmonize research findings across studies, we created narrative summaries based on three general study outcome groupings: 1) pulmonary and ventilatory measures, 2) metabolic and other physiologic measures, and 3) subjective sensations and symptom relief. Moreover, because of the considerable variation in study parameters (e.g., negative vs. positive air ions), study populations (e.g., children with asthma, adult subjects), and outcomes measured (e.g., heart rate, subjective symptoms), we could not justify the combining of data across studies to be evaluated meta-analytically, aside from one exception. We meta-analyzed data from three studies on negative air ion exposure and PEFR [7,13,14].

**Table 1:** Descriptive characteristics of experimental studies on air ions and respiratory outcome measures

**Study author and year**	**Study objective**	**Study design**	**Study population**	**Sample size**	**Primary outcomes of interest**
**Infants**					
[8]	Measure effects of ion exposure to bronchial asthma subjects and comparison to conventional treatment.	Double-blind	Infant patients aged 2–12 months with bronchial asthma.	19 (13 with bronchial asthma and 6 without asthma); 19 additional subjects at different hospital with same diagnosis.	Respiratory rate and scored degree of bronchospasm severity.
**Child-adolescent (up to 20)**					
[6]	Measure therapeutic effect of negative air ions on exercise-or inhaled histamine-induced asthma.	Double-blind randomized	Asthmatic children aged 10–20 yrs recruited from patient population.	11 (for exercise challenge); 9 (for histamine challenge)	FEV1
[15]	Measure efficacy of negative ion treatment for asthma patients.	Double-blind	Asthmatic male students aged 8.8 to 12.6 years at a special school for asthmatics	24	Lung function (whole-body plethysmorgraph and nitrogen washout)
[16]	Measure respiratory effects of positive ions on asthmatic children under physical exertion; follow-up study from [6].	Double-blind randomized	Asthmatic children aged 9–15 yrs recruited from patient population.	12; 7 M and 5 F	Lung function (FEV1 and minute ventilation), oxygen consumption, heart rate, and respiratory heat loss.
[14]	Measure effects of air ions on concentration of airborne dust mite allergen in air and asthmatics	Double-blind crossover	Asthmatic children aged 3–11 yrs recruited from clinic's patient population who's home environments have elevated dust mite allergen air concentration.	20	Peak expiratory flow rate (PEFR) morning and night; self-reported symptom scores; self-medication scores; air concentration of *Der p I* allergen.
**Overlapping child-adult**					
[7]	Measure efficacy of negative ion treatment for asthma patients.	Subject-blind	Asthma patients aged 10–54 yrs; Male=6, Female=1.	7	Peak expiratory flow rate (PEFR); self-reported symptoms.
[17]	Measure efficacy of negative ion treatment for patients suffering from respiratory symptoms	N.S.	Patients male and female aged 7 to 59 years	27	Relief from hay fever, bronchial asthma, neurogenic asthma, acute rhinitis, allergic rhinitis, subacute rhinitis, urticaria, neurodermatitis
[18]	Measure effects of positive and negative ions on hay fever symptoms	N.S.	Patients male and female aged 4 to 59	123	Relief from hay fever and asthma
[10]	Measure physiological and subject effects of breathing ionized air.	N.S.	60 subjects, 25 F and 35 M, aged 10–68 yrs. 45 were normal, 15 had arthritis, 1 had pulmonary tuberculosis, 1 had hypertension, 2 with extreme nervousness, 1 with anemia, and 2 with undernutrition.	60	Pulse rate, blood pressure, respiration rate, mouth temperature, metabolism (oxygen consumption), arterial and finger blood, subjective sensation, subjective impression
[19]	Measure therapeutic effects of negative ions on asthmatics	Double-blind	Chronic asthma patients from hospital aged 15–53 yrs	16	Severity (scored from mild, moderate, or severe) of wheezing, dyspnea, coughing, and septum, and side effects in nose and throat
[20]	Measure pulmonary effects of negative and positive ions.	Subject-blind	Patients (7 F & 8 M) aged 16 to 48 yrs with bronchial asthma who were hospitalized for an extended allergy testing.	15	Lung function (FEV1), histamine threshold for 25% reduction in FEV1, and subjective scoring (air quality, breathing comfort, temperature).
**Adult**					
[21]	Measure physiological effects of negative and positive ions.	Subject-blind	Experiment 1: Six healthy women (age range: 20 to 30 years) chosen at random and Experiment 2: 5 women and 7 men (age range: 19 to 45 years) selected from 125 subjects because they appeared to be most sensitive to ionization	Experiment 1: 6 women Experiment 2: 5 women and 7 men	Experiment 1: skin temperature, rectal temperature, comfort temperature, pulse rate, respiratory rate, mental performance, and subjective feelings of comfort Experiment 2: same as in Experiment 1 except for comfort temperature
[22]	Measure pulmonary, biochemical, emotional, and physical symptom effects of positive and negative ions on asthma.	Double-blind controlled	Asthmatic patients aged 35–64 (8 female and 1 male).	9	Pulmonary (FEV1), pulse and blood pressure, serum theophylline, urinary serotonin metabolite (5HIAA), symptom, response to three questionnaires designed to elicit somatic response and mood changes (Sharav #1 and 2, Adjective check)
[23]	Measure perception of environment, personal comfort, and physical symptom effects of negative ions on workers in a "sick-building" office setting	Subject-blind	Workers in five rooms of office building	26	Linear analogue scores on environment and personal comfort; physical symptom.
[3]	Measure effects on physiological parameter and subjective state from exposure to positive and negative ions.	Subject-blind	Male medical student paid volunteers aged 18–25 yrs; "morning" group N = 6 and "afternoon" group N = 5 to represent different metabolic states during the day.	11	Basal or total metabolism/oxygen consumption (depending on morning or afternoon group), blood pressure, pulse rate, respiratory rate, oral temperature, urine volume, and self-reported subjective state.
[24]	Measure effects of negative ion on physiological parameters and circadian rhythm at rest and during exercise.	Subject-blind cross-over	Male aged 19–25 yrs experienced in physical training and without respiratory ailments.	8	Rectal temperature, heart rate, oxygen uptake (VO2) and minute ventilation (VE), state anxiety per Spielberger (1970), and perception of effort per Borg (1970).
[25]	Measure effects of weather-related positive ions on pulmonary functions of asthmatics	N.S.	6 F and 6 M aged 41–69 yrs recruited from advertisement for subjects with weather-related asthmatic condition	12	Mean peak flow at four times a day measured by subjects using Mini Wright Peak Flow Meter
[26]	Measure physiologic effects and subjective impressions after exposure to light positive and negative air ions.	Subject- blind	17 M and 8 F, aged 22–51 yrs recruited from University research students, lab technicians, and faculty members. Secondary experiments among arthritic patients and infants.	25	Physiological observations such as, heart rate, blood pressure, metabolic rate, respiration; subjective sensations
**Unspecified adult populations**					
[2]	Measure adverse effect of positive air ions and beneficial effect of negative air ions on respiratory allergies.	Double-blind randomized	"Reversible" condition (e.g., hay fever), "partially-reversible" condition (e.g., asthma), and "Irreversible" condition (e.g., pulmonary emphysema) patients; N = 12, 10, and 4, respectively.	26	Six pulmonary functions (VC, total VC1, total VC3, MEFR, MBCR, SBT)
[27]	Measure effects of positive and negative ions on asthmatic, bronchitis, and hay fever patients	N.S.	Patients with mild to moderate asthma, mild bronchitis, or hay fever	24	Lung function (FVC, FEV1, and MMFR)
[28]	Measure pulmonary effects of negative and positive ions.	Not blinded nor randomized	Subjects with severe emphysema/chronic pulmonary disease and/or fibrosis	46; 26; 79	VC, FEV0.5, FEV1, FEV3, MBC, MPFR
[13]	Measure efficacy of negative ion treatment for asthma patients.	Double-blind crossover	Men and women with asthma; 1/20 subject dropped out.	20	Peak expiratory flow rate (PEFR); self-reported symptoms; self medication.
[9]	Measure whether the body is a collector of air ions and biological effects of air ions.	N.S.	77 individuals (half had cardiovascular disease. Various experiments conducted	77	Body as ion collector experiment: electrical current developed between body and ionizer; biological effects study: clinical symptoms (headache, nasal obstruction, husky voice, sore throat, itchy nose, dizziness, congested throat), maximum breathing capacity, and feeling of exhilaration.

N.S.-not specified, FEV-forced expiratory volume, VC-vital capacity, PEFR-peak expiratory flow rate, MBC-Maximum breathing capacity, MPFR-Maximum peak flow rate, MMFR-maximum midexpiratory flow rate; 5HIAA-5-hydroxyindole acetic acid.

Random effects meta-analysis models were used to estimate weighted group mean differences in PEFR, 95% confidence intervals (CI), and corresponding *p*-values for heterogeneity. This type of model assumes that the study-specific effect sizes come from a random distribution of effect sizes according to a specific mean and variance [29]. The group means of the individual studies were weighted based on the inverse of the variance, which is related to the sizes of the study populations. Tests for heterogeneity were conducted and sensitivity analyses were generated to discern any potential sources of between-study variability. Analyses were conducted using Comprehensive Meta-Analysis (version 2.2.046; Biostat, Englewood, NJ).

### Exposure considerations

Some important exposure considerations should be kept in mind in the assessment of the literature on the respiratory effects of air ions. First, except for one or two of the studies reviewed, air ions were generated by concentrating the electric field at the tips of metal needles to produce corona such that the air is ionized and charges are removed and added to gas molecules. Only rarely do studies of air ions consider that this process also generates small quantities of ozone and oxides of nitrogen to varying degrees. In the open air, the concentrations of these gasses to which people might be exposed are vanishingly small, being at the limits of detection even very close to the source [30-32]. The operation of ionizers, however, if not properly designed, can lead to concentrations of these gases that are irritating to the respiratory tract in indoor environments. Indeed, the Food and Drug Administration became involved in the regulation of air ionizers because of concerns about excess ozone production and the lack of a scientific basis for medical device claims in the absence of “well controlled and valid scientific studies” [33,34]. Second, the investigators in these studies also assume that the only exposures produced are to air ions. The lifetime of air ions is quite variable, but generally less than a few minutes in most environments [35]. Many air ions are neutralized by ambient air ions of opposite charge.^2^ Others are neutralized by contact with objects onto which the excess charge is transferred. After neutralization, air ions cease to exist but the charge transferred to aerosols may persist for many minutes or hours. While the essential character of an aerosol is not changed by the addition of electrical charges, it does enhance its susceptibility to forces from other charges. For example, one group of physicists have suggested that when even a single charge is acquired by an aerosol in the size range of 25–125 nm, the deposition of that aerosol on the respiratory tract is enhanced because of the attraction to charges of opposite polarity on its surface [36,37]. Actual studies of the deposition of charged aerosols in human subjects, however, do not support this notion; only when nine or more charges are on such aerosols does deposition begin to increase [38]. Third, all of the experiments reviewed involved the use of air ion generators in indoor laboratory or home settings in which the air ionizers might increase the charge on aerosols above 10 Q per particle [39]; a result that would not occur in well-ventilated rooms or outdoors.

### Summary of studies

The salient characteristics of individual studies including the objectives, study design, population, sample size, and primary outcomes of interest are summarized in Table 1. Table 2 summarizes the ion polarity and concentration of air ions to which subjects were exposed and the study results. The literature on air ion exposure in a controlled environment and respiratory function outcomes spans many decades, with studies published in the English language between 1933 and 1993. Thus, expectedly, the studies vary considerably in terms of study populations being evaluated, experimental design, and outcomes measured, among other factors. Some studies were randomized double-blind experiments, some studies were single blinded or did not incorporate randomization and investigator blinding, and some studies used a cross-over design with variations in experimental methods. The therapeutic effects of air ions, primarily negative polarity, were evaluated in most of the studies. As such, several studies examined the beneficial effect of negative air ions on study populations consisting of children and adults with pre-existing asthma and related respiratory conditions. A wide range of respiratory measures were studied, including respiratory rate, multiple measures of pulmonary function, and respiratory symptoms, after exposure to ionized air particles. Collectively, air ion exposure levels generally between 1,600 ions/cm^3^ and 1,500,000 ions/cm^3^ were measured in the majority of these studies, and the duration of exposure varied considerably across experiments from less than an hour in some studies to weekly intervals. The literature is summarized by general outcome categorizations in the following sub-sections.

**Table 2:** Experimental design and respiratory outcomes

**Study author and year**	**Ion polarity**	**Ion concentration**	**Results**
**Infants**			
[8]	Negative or positive	Calibrated to deliver 1 × 10^4^ ions on 1 cm^2^ area 10 cm away; negative or positive ions.	Negative ion exposure - severity of bronchospasm decreased from 3 to 0.3 after negative ion exposure. Average of 7.5 hrs between start of exposure and effects. Respiratory rate decreased on average 27% after first exposure period. Severity of bronchospasm returned in 7/16 subjects who were followed with cessation of ion exposure; increase in respiratory rate returned in 6/10 subjects who were followed. Positive ion exposure - severity of bronchospasm increased from about 0 to average of 2 after positive ion exposure. Respiratory rate (measured in only 2 subjects) increased on average 20-25% after 3 hrs. Response to positive ion exposure "disappeared spontaneously" after 10 to 50 hrs despite continued exposure. Positive and negative ion exposures - effects are lost when ion concentrations were reduced by a factor of 10 to 20. Control group (at different hospital) - bronchospasm decreased from N.S. to 0 or increased from 0 to 1 after 6 to 7 days of conventional asthma treatment.
**Child-Adolescent (up to 20)**			
[6]	Negative	5 × 10^5^ - 10 × 10^5^ ion/cm^3^; negative; 4 × 10^5^ - 5 × 10^5^ ion/cm^3^; negative	Pre-exercise mean FEV1 before ion exposure 1.36 L/min (SEM 0.07) and after ion exposure 1.35 L/min (SEM 0.08) not significantly different. After exercise challenge mean delta FEV1: Controls 29% of baseline (SE 5%), Exposed 21% (SE 3%) was significantly different (t-test, p<0.015). Histamine challenged mean delta FEV1 before ion exposure was 70% (SE 6%) and after ion exposure was 69% (SE 5%) not significantly different; median provocative dose of histamine was higher with ion exposure than control but difference was not significant, and some patients became more and some less sensitive to histamine challenge after ion exposure.
[15]	Negative	Concentration N.S.; negative	No significant difference in lung function when comparing exposed vs unexposed groups (unpaired t-test) or prior to exposure vs post-exposure for the exposed group (paired t-test).
[16]	Positive	5 × 10^5^ - 10 × 10^5^ ion/cm^3^; positive	Mean delta FEV1 = 35.3% (SEM 5%) with positive ion exposure; 24.7% (SEM 5.3%) control; the difference was significant (paired t-test, p<0.04); other parameters showed no significant change.
[14]	Not specified	N.S.	Difference between active ionizer vs placebo ionizer was significant for airborne allergen concentration (reduction during active ionizer; p<0.0001 Mann–Whitney U-test; p<0.01 Chi-Square test), but non-significant for PEFR, symptom scores, and medication scores. Authors noted increased nighttime cough but difference did not reach a standard significance (p=0.055).
**Overlapping child-adult**			
[7]	Negative	Concentration N.S.; negative	Individual results - Four patient's mean morning PEFR during treatment period significantly improved when comparing to control period (Mann–Whitney U-test; p<0.05). Three patient's mean evening PEFR during treatment period significantly improved when comparing to control period (p<0.01). Three patient's mean morning and evening PEFR significantly decreased when in transition from exposure to control period (p<0.001). Two patients reported subjective improvement during exposure period. Group results - Lung function measurements (from self- and investigator-administered) & diary card scored by investigator showed no significant difference during exposure and control periods (two-way analysis; p>0.4). Lung function measurements (from self- and investigator-administered) alone scored by independent physicians showed no significant difference during exposure and control periods (p>0.7).
[17]	Negative	Concentration N.S.; negative	Hay fever patients (n=17) = 35.3% relief, 47.06% complete relief, 17.64% no relief. All patients (n=27) = 29.63% relief, 33.33% complete relief, 37.04 no relief.
[18]	Negative or positive	Negative 1,200 to 2,600 ion/cm^3^; Positive 2,000-6,500 ion/cm^3^.	Negative ion exposure (n=54) = 62.9% relief; positive ion exposure (n= 5) = 0% relief; control (n = 15) = 6.6% relief. Asymptomatic of hay fever prior to exposure but developed symptoms during exposure: negative ion = 0/37; positive ion = 6/10; control = 1/2.
[10]	Negative or positive	1-50% of generated 5,000-1,500,000 ion/cm^3^; positive or negative	Effects of ion exposure similar regardless of polarity or ion concentration. Tabular summary of averages of measured parameters with ion exposure during basal, 2–4 hrs after breakfast, and 3–5 hrs after lunch. Positive ion exposure resulted in a group of individuals reporting subjective sensation of dryness and irritation of the nose and throat, and frontal headache. Negative ion exposure led to relaxation, and decrease in physiological parameters. Freshness of air felt during negative ion exposure but preference was not strong enough to be significant.
[19]	Negative or positive	N.S. (rate of 1 × 10^-10^ amp); negative and positive	35/40 experiments saw no effects, and 5/40 experiments with negative or positive ion exposure saw mild to moderate wheezing and dyspnea.
[20]	Negative or positive	~30,000 ion/cm^3^; negative or positive.	Group 1 - significant differences in FEV1 over the 4 ion and no ion exposures (Friedman's test, p<0.04). Individual FEV1 higher during both negative (20/27 values) and positive (21/27 values) ion exposure intervals. No significant difference (Friedman's test) in subjective scoring of temperature (p=0.2), air quality (p=0.3), and breathing comfort (p=0.7). Group 2 - no significant difference in histamine threshold after exposure to either ion exposures (Friedman's test, p<0.4) and no change was "demonstrated" (Wilcoxon match pair) in FEV1 from no ion to either positive or negative ion exposure.
**Adult**			
[21]	Negative or positive	300 – 9,000 ions/cm^3^	No significant effects of ionization were observed in either experiment except in certain partial means for the mental performances in Experiment II.
[22]	Negative or positive	60,000 - 110,000 ion/cm^3^; negative or positive	Mean FEV1 and pulse not significantly different between positive and negative ion exposure or from baseline (paired two-tail t-test); blood pressure significantly higher with negative ion exposure (p <0.01; paired two-tail t-test) and after 2 hours of positive ion exposure (p <0.05; paired two-tail t-test); no significant difference between positive and negative ion exposure in serum theophylline, urinary 5HIAA, or in questionnaire results.
[23]	Negative	1841 ion/cm^3^; negative	No significant effects observed, except for slightly more complaints of upper respiratory tract infection and nausea that may have been attributable to mild flu-like disorder in this study population.
[3]	Negative or positive	5 × 10^6^ - 6 × 10^6^ ion/cm^3^; positive or negative	Results for group-level data: basal or total metabolism/oxygen consumption, systolic and diastolic blood pressure = no significant difference between positive, negative, and control exposures. Self-reported subjective state - if 13 comment types are grouped as undesirable or desirable state, "slight difference" (higher) in frequency of reported undesirable state during positive ion than negative ion or control exposures (no statistical comparison shown).
[24]	Negative	172,000 ion/cm^3^; negative ions	With negative ion exposure and at rest, core (rectal) temperature, heart rate, VO2, and VE averaged over four times during the day is reduced significantly in comparison to neutral (no ion exposure) condition (three-way ANOVA, p<0.05). At both 90W and 180W exercise trials, rectal temperatures during the day with negative ion exposure averaged over four times were significantly different from no ion exposure (three-way ANOVA, p<0.05); differences between exposure and no exposure in heart rate (absolute or difference from rest) were insignificant; differences in absolute VO2 and VE between exposure and no exposure were insignificant; differences in the change from rest in VO2 and VE were significant (three-way ANOVA, p<0.05). Differences between exposure and no exposure in the modeled circadian rhythm acrophase amplitude of rectal temperature was significantly during rest (t-test, p<0.05) but not during both exercises. Differences between exposure and no exposure in the modeled circadian rhythm for VO2 and VE were not significant. Differences between exposure and no exposure in perceived exertion during exercise were not significant.
[25]	Negative or positive	Positive and negative ions were measured. Positive ion peak concentration defined as >=2,000 ion/.	Difference between mean peak flow prior to weather fronts and during peak positive ion concentration versus same times during normal days were non-significant (paired t-test).
**Unspecified adult populations**			
[2]	Negative or positive	100,000 ion/cm^3^; negative, positive, or placebo	No significant pulmonary function differences comparing treatments and clinical conditions. Data on grading presented in Figure 1. Results comparing patient grouped by clinical conditions as percentages for each pulmonary function presented.
[27]	Negative or positive	125,000 or greater ion/cm^3^; positive and negative	Mean and S.D. of % change in FVC, FEV1, and MMFR with positive, negative, or control exposure tabulated for each comparison. No significant changes or differences with positive, negative, or no ionization.
[28]	Negative or positive	500,000 ion/cm^3^	1 hour exposure: no significant change with negative ion exposure, 9 subjects; 3 hour exposure: ventilation Factor = 52.2% (S.D. 4.3%) with negative ion exposure; subjectively, 10/33 felt better, 1 worse, and 22 no effect. No correlation between subjective improvement and pulmonary function measurements, 22 subjects; 2 week exposure: ventilation Factor =41.8% (S.D. 5%) with negative ion exposure; subjectively, 10/33 felt better, 1 worse, and 22 no effect. No correlation between subjective improvement and pulmonary function measurements, 15 subjects.
[13]	Negative	±150,000 ion/cm^3^; measured monthly. Group 1 mean = 203,000 ion/cm^3^; Group 2 mean = 183,000 ion/cm^3^.	No significant differences in PEFR, symptom scores, and medication scores were found between active ionizer vs placebo or no ionizer use (paired t-test).
[9]	Negative or positive	Body as ion collector experiment: 32,000 positive ion/cm^3^ or 80% of 32,000 (approx 26,000) negative ions/cm^3^; biological effects study 32,000 ion/ positive or negative.	Biological effects study: Study I-16 with positive ion exposure had symptoms; Study II- 4/13 with negative ion exposures had symptoms; Study III- 2/7 during negative ion exposure and 7/7 during positive ion exposure; Study IV- 3/20 with symptoms during second no ion period, 17/20 with symptoms during positive ion exposure, and 6/20 with persistent symptoms during last no ion exposure period; Study V-1/21 with symptoms during no ion exposure and 5/21 during placebo ion exposure; Maximum breathing capacity study-reduced from 35 L/min to 25 L/min after positive ion exposure, no reduction after negative ion exposure; Effects of grounding study-5/11 developed symptoms with positive ion exposure and grounding, 9/11 developed symptoms with positive ion exposure and no grounding; Temperature and humidity study-no difference in symptoms, during low humidity the symptoms were more frequent and more severe than comparison.

N.A. - not applicable; N.S. - not specified. Forced expiratory volume - FEV; forced vital capacity-FVC; peak expiratory flow rate-PEFR; standard error of mean-SEM; standard deviation-S.D.; 5-hydroxyindole acetic acid-5HIAA; oxygen consumption-VO_2_; minute volume-VE.

### Pulmonary and ventilatory measures

Herrington [3] exposed 11 healthy male volunteers aged 18 to 25 years (6 subjects in the morning group and 5 subjects in the afternoon group) to positive and negative air ions to examine the effects on subjects’ respiratory rate and found that no study participant exhibited significant changes attributed to air ion exposure. The author further confirmed this in a group analysis, whereby no meaningful difference overall in subjects’ respiratory rate was observed. Winsor and Beckett [9] conducted several experimental studies and the overall objectives were to determine if the human body acted as a collector of atmospheric ions and to examine the biologic effects of positive and negative air ion exposure. Only one of their experiments, however, evaluated the respiratory effects of air ion exposure (n = 5 adults). In this study, the maximum breathing capacity (MBC) dropped from 35 L/min to 25 L/min following positive air ion exposure. In contrast, no significant change was observed following negative air ion exposure. Lefcoe [27] evaluated the impact of positive and negative air ion exposure among 24 adults with mild obstructive lung disease (15 mild to moderate asthma patients, 5 mild bronchitis patients, and 4 patients with a history of hay fever) on forced vital capacity (FVC), FEV_1_, and maximum mid-expiratory flow rate (MMFR) measurements. No significant effects on respiratory function between exposure to positive, negative, and no ionization were reported. Blumstein et al. [2] conducted a double-blind randomized study to investigate the influence of positive and negative air ion treatment on allergic respiratory conditions in 26 adults (12 hay fever cases, 10 asthma cases, and 4 pulmonary emphysema cases) and found no significant changes in patients’ conditions when subjectively or objectively assessed by vital capacity, timed vital capacity (TVC_1_ and TVC_3_), MBC, the maximum expiratory flow rate, and the single breath test.

In a cross-over experiment conducted by Reilly and Stevenson [24], oxygen uptake (VO_2_) and minute ventilation (VE) were examined in eight healthy adult males (age range: 19–25 years) exposed to negative air ions. Measurements were taken both at rest and during two consecutive 20-minute sessions of physical activity. The authors observed a significant reduction in mean V0_2_ levels and VE between non-ionized and ionized conditions in resting subjects*.* In contrast, no significant impact of air ions on V0_2_ levels and VE were identified during physical activity. When the authors examined differences between conditions in the delta (exercise minus rest) values of these outcomes, a significant elevation in both V0_2_ levels and VE was noted in the ionized compared to non-ionized conditions.

Motley and Yanda [28] conducted multiple experimental, non-randomized studies among different adult populations to examine the influence of negative and positive air ions on pulmonary function as determined by TVC, FEV, MBC, and mean peak flow rates. In one study of 46 adults with severe emphysema or fibrosis, or both, 13 patients were exposed to negative air ions for 1 hour and 33 patients were exposed to negative air ions for 3 hours, and no significant effect on lung volume measurements were observed. Similarly, the authors reported no significant effect of negative air ion exposure (7 to 12 hours daily for 2 weeks) on lung volume measurements in 19 patients with severe pulmonary emphysema; no significant differences between these 19 patients and 7 unexposed control subjects; and no significant alterations in blood gas exchange measurements (after exposure to negative and positive air ions) or chronic pulmonary disease in 44 and 35 cases, respectively.

Jones et al. [7] performed an experiment during a 16-week period to determine the efficacy of negative air ion treatment for bronchial asthma in seven patients (six males and one female) aged 10 to 54 years. Monthly measurements of lung function included FEV_1_, PEFR, forced mid-expiratory flow, FVC, and static lung volumes. The authors observed that four subjects experienced a significant increase in morning PEFR during the exposure period, but this effect was no longer present in two of these subjects during the subsequent non-air-ion exposure period. In a two-way group analysis, however, they reported that the patients as a whole showed no statistically significant differences between the placebo, treatment, and no treatment periods. Albrechtsen [21] examined pulmonary changes (FEV_1_, histamine threshold) after exposure to positive and negative air ions in 15 patients (8 males and 7 females) aged 16 to 48 years with bronchial asthma. All patients underwent extended allergy testing. In group 1, the researchers identified significant alterations in FEV_1_ between air ion and non-air-ion conditions and individual FEV_1_ levels were significantly greater during both negative and positive air ion exposure periods. Group 2, however, showed no significant change in histamine threshold following air ion exposure and no obvious difference was observed in FEV_1_ levels when subjects were exposed to either positive or negative air ions. The same authors Osterballe et al. [20] reported small, but statistically significant, improvements in lung function in nine of 15 patients with bronchial asthma, and no change in the histamine threshold of the airways in six patients after exposure to ions. Dantzler et al. [22] examined the effect of moderately extended positive and negative air ion exposure in nine adult patients (age range: 35–64 years) with bronchial asthma in a double-blind controlled study, and found that patients’ mean FEV_1_ did not significantly differ between exposures or from baseline.

Nogrady and Furnass [13] evaluated 19 adults (10 men and 9 women, mean age 36 years) in a double-blind crossover study to examine the impact of negative air ion exposure on bronchial asthma. In their 6-month study, the authors found no statistically significant differences in PEFR between active ionization and either placebo or no ionizer environments. Wagner et al. [25] conducted an experimental study to investigate the association between positive or negative air ions, random variations in meteorological factors (ambient temperature, barometric pressure, wind velocity, precipitation, and air pollution), and mean peak flow rates in six male and six female patients (age range: 41–69 years, mean age 54 years) with moderate to severe asthma. The authors found that mean peak flow rates did not differ significantly with alterations in air ion levels or other meteorological parameters linked to the occurrence of two weather fronts during the study.

Palti et al. [8] examined the effects of air ion exposure among 13 infants diagnosed with bronchial asthma and 6 comparison infants free of respiratory symptoms. The authors summarized that negative air ion exposure resulted in reduced respiratory spastic attacks while positive air ion exposure increased spastic attacks in normal infants, however, statistical significance testing was not performed to estimate the reliability of the reported effects. Lipin et al. [16] measured respiratory effects of positive air ions on 12 asthmatic children under physical exertion. Exercise tests were undertaken with and without exposure to positively charged inspired air using a randomized, double-blind design. The authors reported that the post-exercise fall in FEV_1_ was significantly greater (*p* = 0.04) during exposure to positive air ions compared with the control group, but no significant effects were observed for other comparisons (e.g., ventilation, oxygen consumption). In a previous analysis from this study group, Ben Dov et al. [6] evaluated the effect of negative ionization on bronchial reactivity among 11 asthmatic children. The experiment was double-blind and the children were challenged twice by exercise and by histamine inhalation. Exercise induced bronchial reactivity was reduced in all but one study subject, at concentrations of air ions in the mouthpiece approximately 100 to 1,000 times greater than typical background levels. No appreciable effects on resting lung function were observed, and the effect of ionized air on the sensitivity of inhaled histamine was equivocal. In another study of asthmatic boys ages 8 to 12 (n = 24), Kirkham et al. [15] analyzed the effects of negative air ionizers on lung mechanics. They found no significant differences in initial or post-study period lung function values between the groups. Warner et al. [14] evaluated the effect of ionizers on airborne concentrations of house dust mite allergen *Der p I* in a double-blind, crossover, placebo controlled trial. The study was carried out in the homes of 20 children with allergic asthma. Although there was a significant decrease in airborne *Der p I* concentrations, no significant changes were observed for PEFR, symptom scores, or treatment usage. The authors observed a trend in increased night time cough during the active ionizer period, but the association did not reach formal statistical significance.

### Other physiologic measures

The studies included in this review were selected based on their analyses of respiratory effects; however, many of these studies also evaluated other measures as well. Thus, we evaluated other physiological measures in this group of respiratory studies to investigate other potential relationships with air ions. Yaglou et al. [10] performed an experimental study to evaluate metabolic changes (total metabolism, pulse rate, blood pressure, body temperature) during exposure to positive or negative air ions in 60 subjects (25 females and 35 males, age range: 10–68 years) under basal and routine dietary conditions. The study found comparable changes between positive and negative air ion exposure despite the concentration level used, and no noteworthy metabolic alterations attributable to ionization were identified. In a subsequent experimental study conducted by Yaglou [26], 25 healthy adults (17 males and 8 females, age range: 22–51 years) were exposed to positive or negative air ions for 1 to 2 hours in between pre- and post-test control periods. No significant differences in subjects’ metabolic rate, blood pressure, oral temperature, and red and white blood cell counts were found. The authors also conducted an experiment in six arthritic adult patients exposed to positive or negative air ions and observed no major changes in metabolism, heart rate, and blood pressure, except in anxious patients experiencing air ion treatment for the first time. In addition, they examined if negative air ion therapy was beneficial to the growth and development of five infants, and found that babies’ weight gain, heart rate, and body temperatures did not significantly change when exposed for 2 hours during a 2 week ionization period compared to non-ionization periods.

Summarized in the previous pulmonary section, Motley and Yanda [28], Dantzler et al. [22], Reilly and Stevenson [24], Herrington [3], and Lipin et al. [16] also examined metabolic parameters. Motley and Yanda [28] reported the pulse rate per minute between positive and negative air ion exposure in their blood gas exchange study (n = 44) and found that the average pulse rate was slightly lower when exposed to negative versus positive air ion therapy (77 vs. 81) but the authors did not conduct statistical significance testing. The Dantzler et al. [22] double-blind controlled study of nine adult patients showed no significant changes in the elimination of catecholamine metabolites or in pulse rate between positive and negative air ion exposures, but reported that mean blood pressure rose significantly between baseline and 2 hours of positive air ion exposure. In the cross-over study of eight healthy adult males performed by Reilly and Stevenson [24], negative air ion exposure resulted in statistically significant decreases in rectal temperature, heart rate, and metabolic rate at rest; however, no effects on metabolism and heart rate remained while subjects exercised. In the aforementioned experimental study conducted by Herrington [3], no study participant exhibited significant changes in basal or total metabolism, blood pressure, pulse rate, oral temperature, and total urine volume that were attributable to air ion exposure. Furthermore, no meaningful group differences in metabolic rate or blood pressure were observed. In a randomized, double-blind study of 12 asthmatic children, no significant differences were observed for heart rate or respiratory heat loss after exposure to positive air ions [16].

### Subjective sensations and symptom relief

In the earlier Yaglou et al. [10] study discussed previously, the most prevalent sensation effects reported in the positive air ion experiments were dryness and irritation of the nose (13.5%), headache (13.5%), and an invigorating, stimulating sensation (10.8%), while others reported feeling no change (21.7%). On the other hand, the most prevalent sensations reported in the negative air ion experiments were relaxation (21.6%), a general cooling effect (12.9%), and sleepiness (12.9%), while a group reported feeling no change (27.6%). In their later experiment [40], Yaglou reported that negative air ion exposure did not impact subject’s perception of the quality of the air of 25 adult subjects, although positive air ion exposure appeared to increase upper respiratory tract irritation. The author noted that the majority of the experiments were conducted during the winter, when such sensations were more prevalent. In addition, reported joint symptoms did not improve when the arthritic patients under study were exposed to negative air ion therapy, while positive air ion exposure appeared to result in unfavorable symptomatic effects. The extremely small sample size greatly limits any possible inferences that could be made, however.

Zylberberg and Loveless [19] conducted a double-blind, controlled study on 16 asthmatic men and women (aged 15–53) during two 120-minute exposure periods to ionized air. No differences in the biologic effect of positive or negative air ions were observed, although dryness of the nose or throat was reported for both ion polarities. Kornblueh and Griffin [17] measured the efficacy of negative air ion treatment among an adult and child patient population (n = 27) who suffered from respiratory symptoms. The majority of patients were previously diagnosed with hay fever, while a few were diagnosed with asthma or variants of rhinitis. The authors indicated that the majority of subjects reported complete or partial relief for hay fever symptoms, but there was no appreciable effect for patients with asthma or rhinitis. In a subsequent publication by Kornblueh and colleagues [18], the effects of positive and negative air ions on hay fever symptoms were evaluated among 123 children and adults aged between 4 and 59. Exposure to negative air ions was associated with hay fever symptom relief among symptomatic subjects, but did not result in symptoms among asymptomatic subjects. Positive air ion exposure did not result in symptom alleviation, but was associated with the development of symptoms in asymptomatic subjects. Of note, the sample size of the positive air ion group was considerably smaller than the negative air ion group. Statistical testing was not performed. In the Dantzler et al. [22] study previously discussed, no statistically significant differences in reported somatic symptoms among eight study participants were observed between positive and negative air ion exposures. In a double-blind, crossover, placebo controlled trial of ionizers in the homes of asthmatic children, Warner reported no significant differences between groups for night/day wheeze, night time cough, or daytime activity [14].

“Sick building syndrome” has been described as discomfort within office buildings, and a deficiency of negative air ions has been hypothesized as contributing to symptoms. Thus, Finnegan et al. [23] conducted a survey in a “sick building” whose occupants had a high prevalence of symptoms to test for beneficial effects of negative air ion generators. Twenty-six subjects completed a questionnaire daily for 12 weeks to rate the environment and their physical comfort. There were no significant effects on environment or personal comfort factors. There were slightly more complaints of upper respiratory tract infections and nausea, but these may have been attributable to mild flu-like disorder.

### Meta-analysis of PEFR

**Figure 1:**
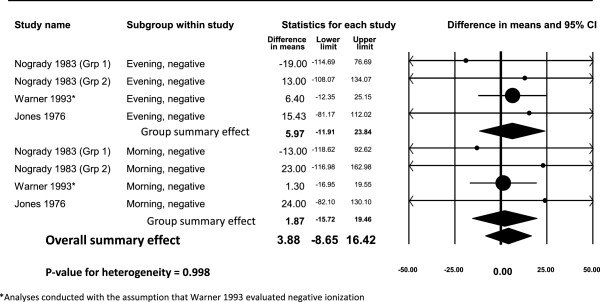
Difference in group means for PEFR (L/min) testing after exposure to negative air ions.

We were able to combine data from three studies (eight unique parameter estimates) in a meta-analysis that evaluated negative air ion exposure and PEFR [7,13,14]. The studies reported group mean values for PEFR in the morning and evening (Figure 1). The weighted difference in group means (i.e., PEFR after negative air ion exposure [post-test]; PEFR before negative air ion exposure [pre-test]) for the morning testing was 5.97 but this difference was not statistically significant (95% CI: -11.91 – 23.84). For the evening testing, the weighted difference in group mean values was attenuated and also not statistically significant (1.87, 95% CI: -15.72 – 19.46). When data for both morning and evening tests were combined, the weighted difference in group mean values was 3.88 (95% CI: -8.65 – 16.42) with virtually no statistical heterogeneity present (*p*-heterogeneity = 0.998). Blumstein et al. [2] reported group results for the maximum expiratory flow rate (L/min) in a bar chart, but did not report actual group data. Based on their bar chart, there does not appear to be an appreciable difference between group mean values comparing negative ionization with the control group. Overall, the meta-analysis findings were not supportive of a statistically significant effect of negative air ion exposure on PEFR measures.

## Discussion

Over several decades, the effects of artificially generated air ions on humans have been studied for both experimental and therapeutic purposes, and attempts have been made to investigate naturally occurring variations in air ion levels in relation to a variety of physiological conditions. To our knowledge, this is the first comprehensive review to summarize human studies of air ion exposure and respiratory outcomes other than those that were designed to test for potential therapeutic effects. Air ions are simply air molecules that have gained or lost electrical charges based on the displacement of an electron from a neutral gas molecule. In terms of physiological aspects, the interactions of air ions with the body are similar to interactions with other components of the air, such as oxygen and nitrogen, except that charged molecules and atmospheric aerosols carrying charges can be attracted to and deposited on the skin and respiratory tract by electrostatic forces. In regard to the respiratory tract, most of the air ions are retained in the nose and bronchi with few reaching the deep alveoli of the lung [12]; however, no mechanism has been established or confirmed to explain how air ions could exert any significant biological effect on respiratory or other systems [12]; NRPB, [41]. This is not surprising when one considers that even 100,000 ions represent an infinitesimal concentration in the air (100,000/10^19^ molecules in 1 cm^3^). Should air ions be deemed toxic, the threshold for effect would be lower than some of the most potent toxins (e.g., botulism) [11]. In fact, no scientific or regulatory agency has determined that small air ions pose a threat to the environment or health and no exposure guidelines have been proposed. The only guidelines for air ions have been published by the Ministry of Health of the Russian Federation for maintenance of optimal levels in indoor environments (i.e., maintaining levels of air ions at or above levels in clean outdoor air) because low levels of air ions in buildings have been alleged as symptomatic of poor indoor air quality [MHRF, [42]].

Synthesizing and examining the scientific evidence on a topic such as this is a challenging undertaking, which is complicated by the considerable variation in experimental methodology, study populations being evaluated, and differing outcome measures. A major strength across the majority of studies is the controlled experimental design, whereby the investigators or study participants, or both, may have been blinded to the exposure (i.e., ion polarity) parameters. In addition, the random allocation of subjects to exposed and control groups theoretically reduces the confounding influence of extraneous factors. Not all studies utilized blinding or randomization techniques, however, and approximately half of the studies examined sample sizes of less than 20, potentially resulting in diminished statistical power to observe a statistically significant effect in these studies. For example, the studies did not control for the reduction in particulate levels by air ionizers, and if a beneficial effect was reported, the result may have been due to the reduction of particulate levels, such as dust or allergens, in the room. Across studies, there is considerable variation in the way outcome information and data were analyzed, reported, and tested for significance. This heterogeneity may be due, in part, to the varying levels of scientific rigor and sophistication of statistical techniques available, given the expansive time frame and historical context in which the studies were published. For example, some studies simply reported data using graphical illustrations, some reported group averages, some reported clinical parameters for selected subjects, and some did not report data. In addition, the utilization of significance testing varied as did the reporting of variance data, such as standard deviations or confidence intervals. The lack of uniformity in terms of exposure factors (e.g., positive vs. negative air ions, group mean change in respiratory function vs. individual effect), outcome measure (e.g., PEFR, body temperature), and data reporting limits the feasibility to conduct a quantitative evaluation of the available literature, such as a meta-analysis.

Meta-analyses are becoming more and more prevalent in the peer-reviewed literature, and serve as a useful tool in weight-of-evidence evaluations and public policy and regulatory decision making. An important function of a meta-analysis is to estimate the collective strength of an association, examine the consistency of study findings, identify potential sources of between-study heterogeneity, and appraise the likelihood of publication bias. Although numerous studies on air ion exposure and respiratory outcomes have been published, as mentioned, considerable variation (e.g., study population differences, positive vs. negative polarity) across studies exists, precluding a formal comprehensive quantitative assessment. We were, however, able to combine data on negative air ion exposure and PEFR in a meta-analysis. This analysis indicated slight improvement in PEFR after exposure to negative air ions but the effect was not statistically significant. To more appropriately explore collective quantitative evaluations on air ion exposure and respiratory outcomes, any future studies should transparently document all analytical and statistical methods and data to facilitate a more uniform comparison of findings across studies. Indeed, in the aforementioned Cochrane Collaboration publication of effectiveness of positive and negative air ion generators among persons with asthma, the authors indicated that they could not reliably pool data together across studies [1].

Despite the limitations indicated above, the experimental studies reviewed here provide no persuasive evidence for an effect of charged air ions on respiratory effects, including pulmonary and ventilatory measures (Table 3), metabolic and physiologic parameters, and subjective symptom alleviation and sensations. This interpretation is largely based on fundamental factors that include the strength of effect and whether any effect is statistically significant and free from bias, confounding, or chance; evidence of dose–response relationships; and consistency of findings across studies. Collectively, in the majority of studies, the effects were relatively weak in magnitude (irrespective of the outcome evaluated), inconsistent as to the direction of the response, and not indicative of a dose–response trend. This observation is in concert with the aforementioned MEQB review, which stated that only minor symptoms (e.g., throat dryness) were related to experimental air ion exposures, with limited evidence of any dose–response relationships [12]. Further, in the MEQB review it was reported that short- and long-term exposures to positive and negative air ions do not affect persons with pre-existing allergies, asthma, or respiratory disease, or persons more sensitive to respiratory irritants. As mentioned, Blackhall et al. [1] also concluded that research has failed to demonstrate any benefit of air ionizers in the treatment of chronic asthma in children and adults.

**Table 3:** Reported overall study conclusions for air ions and pulmonary and ventilatory measures

**Study**	**Ion polarity evaluation**			**Conclusions reported in article**
	**Negative**	**Positive**	**Both**	
[27]			**X**	“It is concluded that we have not shown any effect of highly ionized air upon these ventilator tests.”
[6]	**X**			“It is concluded that negative ionization of inspired air can modulate the bronchial response to exercise but the effect on the response to histamine is much more variable.” [Note: no effect seen in non-exercise challenge]
[16]		**X**		“It is concluded that positive ionization aggravates the bronchial response to exercise.” [Note: only significant difference was for post-exercise FEV comparisons]
[22]			**X**	“…exposure to positive or negative small air ions did not influence the clinical condition…findings do not support a significant role of small air ions in exacerbation or treatment of bronchial asthma.”
[21]			**X**	“A slight but significant (at the 5% level) improvement in the lung function was demonstrated during positive as well as negative ion exposure…”
[20]			**X**	“A slight but significant (at 5% level) improvement in the lung function was demonstrated in nine patients during positive as well as negative ion exposure…”
[28]			**X**	“No significant changes were observed in the lung volume measurements…after breathing the negative ions.” [Note: no effect for short or long exposure] “To date our work has failed to demonstrate any significant objective changes which can be measured from breathing of negative or positive ions either favorable or unfavorable.”
[13]	**X**			“There were no significant differences in PEFR…between the periods that active ionizers and either no ionizers or placebo ionizers were in operation…study has failed to show a statistically significant benefit in asthmatic subjects from the use of negative ion generators.”
[7]	**X**			“…it is unlikely that exposure to negative ions will be of significant benefit in the majority of patients with asthma…the effects of negatively ionized air on such patients remains to be determined.”
[2]			**X**	“…failed to show any significant effects when judged by subjective clinical appraisal or evaluated by objective pulmonary function…ionization should not be recommended as a therapeutic adjuvant in the treatment of these diseases.”
[25]		**X**		“The mean peak flow rates in this group of patients did not vary significantly with the changes in ion levels or other meteorologic factors which resulted from the passage of these weather fronts.”
[14]	X*			“This study indicates that the use of ionizers cannot be recommended in the homes of asthmatic subjects to improve their symptoms.”
[10]			**X**	“No significant changes were found in…exposures of between one and two hours to either positive or negative ions, compared to changes which occurred in control experiments.” “As in our previous work, nothing definite was found to justify the use of artificial ionization in ventilation or air conditioning.” [Note: upper respiratory irritation increased after exposure to positive ions, based on subjective responses but may be due to weather effects.]
[9]			**X**	“…positive ions produce irritation of the respiratory tract especially when the humidity is low, the patient is grounded and high ion densities are employed.” [Note: primarily based on subjective symptom responses, not objective clinical measurements.]
[10]			**X**	“…under the conditions of the present experiments nothing definite was found to justify the use of artificial ionization in general ventilation.”
[3]			**X**	“They [the experiments] certainly do tend to justify the opinion that, so far as normal subjects are concerned, such effects are unproven and improbable.”
[8]			**X**	“It was demonstrated that atmospheric ions have an effect on infants, especially those suffering from asthmatic (spastic) bronchitis.” [Note: in some subjects, negative ions had a beneficial impact on bronchial spasms and respiration rate, and positive ions had a deleterious impact in spastic attacks in some normal infants.]
[15]	**X**			“We concluded that nocturnal administration of negative air ionization has no significant effect upon lung function in the asthmatic child using the above tests.”
[24]	**X**			“…negative air ions significantly reduced resting values of all physiological variables…these effects tended to disappear under exercise conditions.”
[19]			**X**	“…no difference in the biologic effect of positive and of negative atmospheric ions…the negative (like the positive) ions did not appear to influence the patient’s typical pattern of wheezing and remission.”
[17]	**X**			“…twenty-seven patients were exposed to the influence of negative ionization in an experimental room. Many patients with hay fever and asthma responded favorably to the physical alteration of the environment.”
[17]			**X**	“Favorable responses were elicited by the negative polarity. Positive ionization caused either no relief or increased distress.”
[23]	**X**			“Negative ion generators are not to be recommended for this problem [sick building syndrome], especially as the data on temperature and humidity provided a good 'internal control' that real effects were being measured.”

*not stated explicitly-presumed negative.

Based on the constellation of literature spanning numerous decades and in light of variations in experimental study designs, study populations, outcome measurements, and analytical techniques, exposure to negative or positive air ions and any associated exposures to charged aerosols does not appear to play an appreciable role in respiratory function. Although some studies have reported a variety of pulmonary benefits after exposure to negatively charged air ions, and some studies have reported a few mildly unfavorable pulmonary responses after exposure to positively charged air ions, collectively, the literature does not provide any reliable evidence for effects of negative or positive air ions on pulmonary, respiratory, or metabolic measures.

## Endnotes

^1^An examination of English abstracts of studies published in foreign languages did not suggest conclusions different from those based on studies published in English.

^2^About 1/3 of aerosols are positively charged, 1/3 negatively charged, and 1/3 without charge in a Boltzman equilibrium [NRPB, [41]].

## Competing interests

The authors declare that they have no competing interests.

## Authors' contributions

DDA conducted the analyses and contributed to the writing; VP, MEM, SS contributed to the writing and reviewed the manuscript; WHB contributed to the writing, review, submission and oversight of the manuscript. All authors read and approved the final manuscript.
